# Astrocyte Transcriptomics in a Three-Dimensional Tissue-Engineered Rostral Migratory Stream

**DOI:** 10.3390/cells14211646

**Published:** 2025-10-22

**Authors:** Michael R. Grovola, Erin M. Purvis, Andrés D. Garcia-Epelboim, Elizabeth N. Krizman, John C. O’Donnell, D. Kacy Cullen

**Affiliations:** 1 Center for Neurotrauma, Neurodegeneration & Restoration, Corporal Michael J. Crescenz VA Medical Center, Philadelphia, PA 19104, USA; mgrovola@pennmedicine.upenn.edu (M.R.G.); epurvis@uci.edu (E.M.P.); garciaan@sas.upenn.edu (A.D.G.-E.); elizabeth.krizman@pennmedicine.upenn.edu (E.N.K.); 2 Center for Brain Injury & Repair, Department of Neurosurgery, Perelman School of Medicine, University of Pennsylvania, Philadelphia, PA 19104, USA; 3 Department of Neuroscience, Perelman School of Medicine, University of Pennsylvania, Philadelphia, PA 19104, USA; 4 Department of Physics and Astronomy, School of Arts and Sciences, University of Pennsylvania, Philadelphia, PA 19104, USA; 5 Department of Bioengineering, School of Engineering and Applied Science, University of Pennsylvania, Philadelphia, PA 19104, USA

**Keywords:** astrocytes, transcriptomics, rostral migratory stream, tissue engineering

## Abstract

The glial tube is a longitudinal structure predominantly composed of densely bundled, aligned astrocytes that projects from the subventricular zone (SVZ) to the olfactory bulb. Neural precursor cells (NPCs) generated in the SVZ migrate through this glial tube—referred to as the rostral migratory stream (RMS)—to replace olfactory bulb interneurons in the mammalian brain. RMS astrocytes have distinct morphological and functional characteristics. These characteristics facilitate the unique purpose of the RMS as an endogenous living scaffold directing NPC migration and maturation. However, the transcriptomic factors underlying these unique structure–function attributes versus standard stellate astrocytes have not been examined. We previously developed biofabrication techniques to create the first tissue-engineered rostral migratory stream (TE-RMS) that replicates key features of the glial tube in vivo. We have shown that TE-RMS astrocytes exhibit elongated nuclei, longitudinally aligned intermediate filaments, and enrichment of key functional proteins—cytoarchitectural and surface features characteristic of native RMS astrocytes. In the current study, we performed RNA-seq on TE-RMS astrocytes in comparison to planar astrocyte cultures to identify gene expression patterns that may underlie their profound morphological and functional differences. Remarkably, we found 4,008 differentially expressed genes in TE-RMS astrocytes, with 2076 downregulated (e.g., LOC690251 and *ccn5*) and 1932 upregulated (e.g., *lrrc45* and *cntn1*) compared to planar astrocytes. Moreover, there were 256 downregulated and 91 upregulated genes with >3-fold change. We also conducted analyses of gene sets related to cytoskeleton and nuclear structure, revealing the greatest enrichment of actin-related components. Overall, the TE-RMS offers a platform to study the interplay between transcriptomic and cytoarchitectural dynamics in a unique astrocyte population.

## 1. Introduction

Astrocytes are the most abundant cell type in the mammalian brain and are critical to maintaining homeostasis due to myriad roles, including providing metabolic, trophic, and physical support for neurons, controlling the blood–brain barrier, and regulating the synaptic microenvironment [1,2,3,4]. Astrocytes derive their name from their stereotypical star-shaped morphology and are generally distributed homogeneously in non-overlapping domains throughout the brain [1,5,6]. In contrast, the glial tube is composed of a curious astrocyte subtype marked by aligned, bidirectional, interwoven processes in a dense, rope-like bundle [7,8,9,10]. The glial tube projects longitudinally from the subventricular zone (SVZ) to the olfactory bulb and plays a key role in guiding the migration, maturation, and differentiation of new neurons [11,12,13,14].

In particular, the formation of new neurons in the brain, also known as neurogenesis, occurs in the SVZ and continues throughout adulthood in most mammals [15,16]. In the SVZ, neural precursor cells (NPCs) are generated and can differentiate into neuroblasts that migrate long distances along the glial tube—referred to as the rostral migratory stream (RMS)—to the olfactory bulb, where they integrate into the existing olfactory circuitry as various types of interneurons [13,14,17,18]. Neuroblasts can be diverted from the RMS pathway towards injured brain regions, guided by chemoattractive factors, and can mature into functional neurons of a region-specific phenotype [19,20,21,22,23,24]. Furthermore, functional recovery was found after experimentally enhancing the delivery of neuroblasts from the SVZ into injured brain regions [23,24,25,26,27,28,29,30,31,32,33]. Our research team and others have augmented this redirection of neuroblasts utilizing a variety of tissue engineering and biomaterial strategies [34,35,36].

Specifically, our team developed the first tissue-engineered RMS (TE-RMS), a living, biologically-active scaffold comprising aligned, bundled astrocytes that mimics the endogenous RMS. The TE-RMS has been designed to facilitate the migration of NPCs to target brain regions [9,10,11,12,37,38]. Our previous experiments have demonstrated that the TE-RMS can be fabricated from rat astrocytes or human astrocytes derived from mesenchymal stem cells that naturally exist in the gingiva, that TE-RMS astrocytes express key functional proteins of the endogenous rat RMS (e.g., ezrin and robo2), and that they facilitate migration of immature rat cortical neurons in vitro as well as in vivo post-implantation [12]. Recently, we have also demonstrated that the TE-RMS can direct migration of neuroblasts specifically harvested from the rat subventricular zone [38]. By structurally and functionally mimicking the endogenous RMS, TE-RMS implants may create a migration pathway to injured brain tissue, and thus provide a mechanism for gradual yet sustained neuronal replacement as a regenerative medicine strategy.

**Figure 1:**
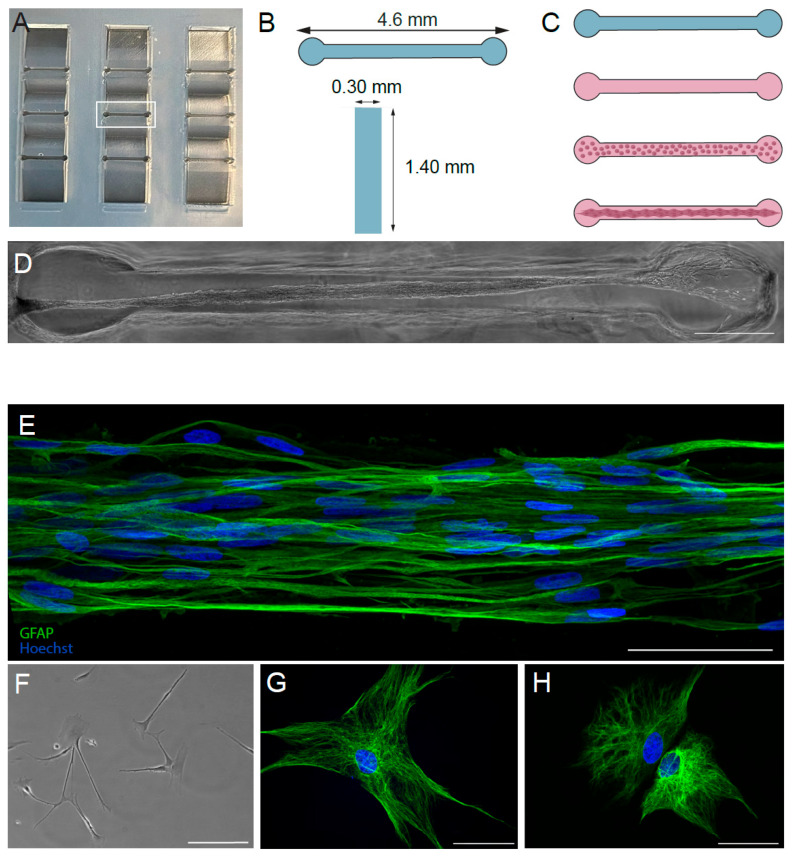
Fabrication and architecture of tissue-engineered rostral migratory streams. TE-RMSs were fabricated in 3 × 3 grids of hydrogel rectangular microchannels (**A**). The central microchannel in the 3 × 3 grid is squared in white (**A**). Microchannels were 4.6 mm long, 0.3 mm wide, and 1.4 mm deep (**B**). To fabricate TE-RMSs, microchannels were dried and coated with extracellular matrix. Following complete polymerization and drying of the extracellular matrix, microchannels were seeded with a dense suspension of astrocytes that bundled into TE-RMSs (**C**). An example of a bundled TE-RMS in a microchannel is shown under phase microscopy (**D**). High-magnification confocal imaging reveals the structure of TE-RMS astrocytes with elongated cell nuclei and bidirectional intermediate filament processes (**E**). Astrocytes are labeled in green with an anti-glial fibrillary acidic protein antibody, and all nuclei are labeled in blue with Hoechst. TE-RMS astrocytes (**D**,**E**) have a distinct architecture compared to planar astrocytes (**F**–**H**). Planar astrocyte samples visible under phase (**F**) and fluorescence microscopy (**G**,**H**) depict astrocytes with round nuclei and intermediate filament processes spread in all directions. Scale bars: 500 µm (**D**), 20 µm (**F**), and 50 µm (**E**,**G**,**H**).

TE-RMSs are fabricated within hydrogels using geometric cues (e.g., hollow microcolumns or microchannels) and an extracellular matrix coating (Figure 1A,B) [9,10,11,12,36,38]. This fabrication process induces the self-assembly of dissociated (spherical) astrocytes into three-dimensional, longitudinally aligned bundles (Figure 1C). We have previously shown that the TE-RMS replicates the structure and key protein expression of the endogenous rat RMS. We have also quantified the extent of morphological changes in the bundled, longitudinally aligned TE-RMS astrocytes versus astrocytes in typical planar culture. Specifically, cytoskeletal rearrangement and nuclear elongation were clearly evident in TE-RMS astrocytes, similar to RMS astrocytes in vivo [37]. Relatively recent scientific advances have identified molecular mechanisms initiated by mechanical stress on the cytoskeleton, which may alter nuclear structure and chromosome dynamics [39,40]. These cytoarchitectural adaptations, as cells move from two-dimensional environments into three-dimensional environments, are necessary for the cell body and nucleus to reshape, allowing, for instance, cell migration through the available space [41].

It is evident that RMS astrocytes have distinct morphological and functional characteristics, facilitating their unique purpose as an endogenous living scaffold directing NPC migration and maturation. However, the transcriptomic factors underlying these unique structure–function attributes versus standard stellar astrocytes have not been examined. Accordingly, in the current study, we conducted high-throughput RNA sequencing on TE-RMSs to identify gene expression changes that may have contributed to or resulted from morphological changes and compared expression levels to planar astrocytes in vitro. Then, we performed gene set analysis on annotated gene sets related to cytoskeleton and nuclear structure. We hypothesized that TE-RMS astrocytes would have significant changes to cytoskeletal and nuclear-related genes compared to planar astrocytes due to their altered cytoarchitecture. This study provides insight into the transcriptomic and structural responses of astrocytes following TE-RMS formation. Moreover, this study highlights the TE-RMS as a novel platform to investigate cytoskeletal arrangement and nuclear morphology, while also identifying genetic targets to customize astrocyte populations for potential utility in regenerative applications.

## 2. Materials and Methods

### 2.1. Isolation and Culture of Astrocytes

**Figure 2:**
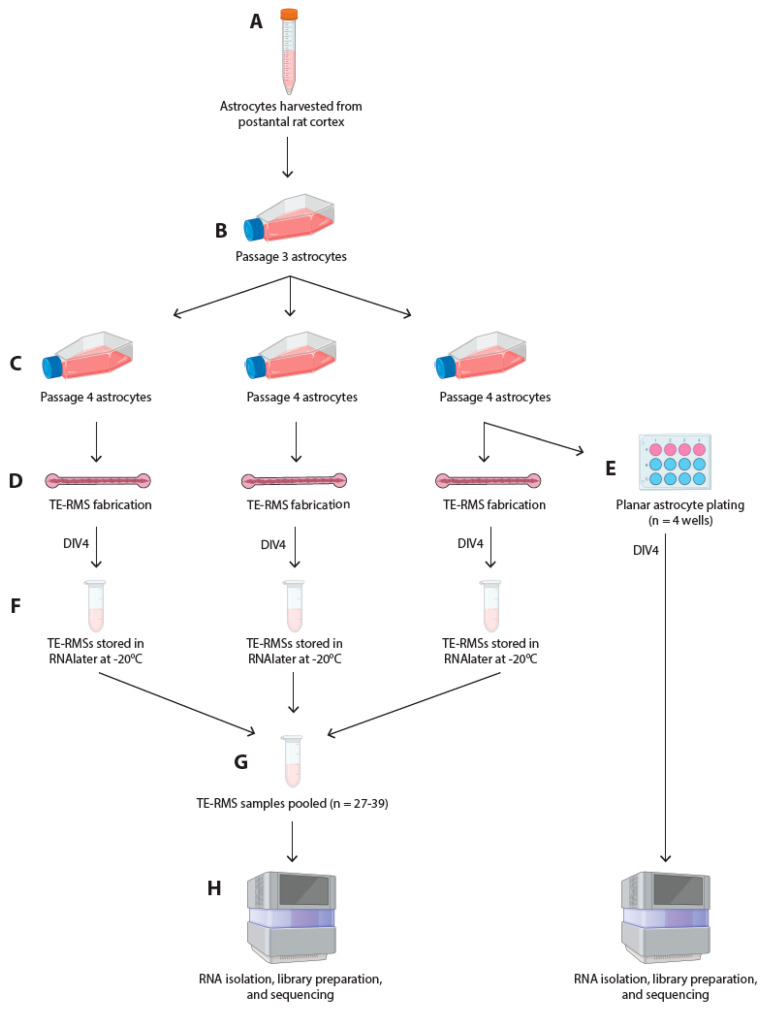
Experimental timeline. Astrocytes were harvested from the postnatal day 0–1 rat cortex (**A**). Cells were cultured in T75 flasks and purified through passaging. Passage 3 astrocytes (**B**) were split into 3 separate passage 4 sister flasks (**C**). Three sequential TE-RMS fabrication days (**D**) were required to generate a sufficient quantity of TE-RMSs for RNA sequencing, with 1 T75 flask required for each fabrication day. On the third sequential day of TE-RMS fabrication, planar astrocytes were also plated in 4 wells of a 12-well plate (**E**). At DIV4, following TE-RMS fabrication, bundled TE-RMSs were extracted from their microchannels, placed in RNAlater, and stored at −20 °C (**F**). Preserved TE-RMSs were pooled into a single sample (**G**). RNA was simultaneously extracted from DIV4 TE-RMS samples (frozen; n = 27–39 TE-RMSs) and DIV4 planar astrocyte samples (fresh; n = 4 wells) (**H**). The entire process represented in this flow chart was repeated 3 times (defined as Run 1, Run 2, and Run 3), facilitating RNA sequencing on 3 distinct TE-RMS and 3 distinct planar astrocyte samples. Astrocytes were harvested from different animals for each repetition.

All procedures described herein adhere to the National Institutes of Health Guide for the Care and Use of Laboratory Animals and were approved by the Institutional Animal Care and Use Committee at the University of Pennsylvania. Primary cortical astrocytes were harvested from postnatal day 0–1 Sprague–Dawley rat pups (female or male) (Figure 2A). Following dissociation [described previously in [10]], astrocytes were cultured in Dulbecco’s Modified Eagle Medium F12 (DMEM/F12; Gibco, Thermo Fisher Scientific, Waltham, MA, USA; #11330032) supplemented with 10% Fetal Bovine Serum (MilliporeSigma, Burlington, MA, USA; #F0926) and 1% Penicillin–Streptomycin (Gibco #15140122) antibiotics in a cell culture incubator maintained at 37 °C and 5% CO_2_. Astrocyte flasks were passaged with trypsin–EDTA (Gibco #25200056) at 80% confluency to maintain the astrocyte cell lines. Passage 3 astrocytes were split into 3 separate passage 4 sister flasks (Figure 2B,C). Astrocytes at passage 4 were used for TE-RMS fabrication and planar astrocyte plating (Figure 2C–E).

### 2.2. Fabrication of Molds and Hydrogel Microchannels

Fabrication of molds and hydrogel microchannels has been recently described in detail by our group [38]. Briefly, microchannels were fabricated with custom stamps containing 9 channels arranged in a 3-by-3 grid, with wells to connect media away from the channels (Figure 1A). Channels were 4.6 mm long, 0.3 mm wide, and 1.4 mm deep (Figure 1B). The desired channel design was reversed to create printable stamps that would create the channels when stamped into agarose. Stamps were printed in high-temperature resin at the University of Pennsylvania Libraries’ Holman Biotech Commons. Following printing, stamps were carefully removed from their supports, cleansed with deionized water, and autoclaved. Agarose (Sigma #A9539-500G) and phosphate-buffered saline (Gibco #14190136) were mixed to create a 3% weight-by-volume agarose solution that was boiled until it was completely transparent and devoid of bubbles. The agarose was pipetted into 60 mm sterile dishes and spread evenly across the bottom of the dishes prior to stamps being placed into the hot agarose. Once the agarose had hardened, the stamps were removed. Three milliliters of PBS was added to each dish to keep the molds hydrated.

### 2.3. Fabrication of Tissue-Engineered Rostral Migratory Streams and Planar Astrocyte Cultures

Fabrication of TE-RMSs in hydrogel microchannels has been recently described in detail by our group [38]. Briefly, PBS was removed from microchannel molds using a glass Pasteur pipette and vacuum. Channels (Figure 1A) were seeded with cold rat tail type 1 collagen (Advanced BioMatrix, Carlsbad, CA, USA; #5153) diluted in a neutralization buffer containing 50% TE-RMS culture media [a base of Neurobasal media supplemented with 2% B27 (Invitrogen, Thermo Fisher Scientific, Waltham, MA, USA; #12587010), 0.25% L-glutamine (Gibco #35050061), 1% G5 supplement (Gibco #17503012), and 1% Penicillin–Streptomycin (Gibco #15140122)], 14.1% cell culture water (Corning Inc., Corning, NY, USA; #25055CM), 0.5X minimum essential media (Gibco #11430030), 25 mM HEPES (Gibco #15630080), 26.2 mM NaHCO_3_ (Gibco #25080094), and 0.5X G5 supplement (Gibco #17503012). Dishes were placed in an incubator at 37 °C and 5% CO_2_ for around 2 h until the collagen completely polymerized to coat the inner walls of the microchannels. During this collagen polymerization period, a flask of 80% confluent astrocytes was passaged with 0.25% trypsin–EDTA (Gibco #25200056), and cells were re-suspended in TE-RMS culture media at a density of 2.5 million cells/mL. Following complete collagen polymerization in the microchannels, astrocytes were seeded into the channels, and dishes were returned to incubate at 37 °C and 5% CO_2_. One hour later, dishes were flooded with TE-RMS culture media and returned to incubate at 37 °C and 5% CO_2_. Under these conditions, astrocytes bundled together with collagen to self-assemble into TE-RMSs (Figure 1C–E). Three sequential TE-RMS fabrication days were required to generate a sufficient quantity of TE-RMSs for RNA sequencing, with 1 T75 flask required for each fabrication day (Figure 2C,D). TE-RMSs at DIV4 after plating were used for all experiments (Figure 2D,F). For planar astrocyte samples, flasks of 80% confluent astrocytes were passaged with 0.25% trypsin–EDTA (Gibco #25200056). Cells were then resuspended in TE-RMS culture media and were plated on top of polymerized 1 mg/mL collagen in 12-well plates at a density of 2.5 million cells/mL in TE-RMS culture media. Planar cultures at DIV4 after plating were used for all experiments (Figure 2E).

### 2.4. Immunocytochemistry

For fluorescent imaging, cultures were fixed with 4% paraformaldehyde for 30 min at room temperature. Planar astrocyte samples were fixed and stained directly on the coverslips on which the cells were grown. For TE-RMS samples, a glass coverslip was coated with 0.002% poly-l-lysine (Sigma #P4707), incubated for 2 h at 37 °C, rinsed 3 times with cell culture water (Corning #25055CM), and left to dry. A small quantity of freshly made collagen (see section detailing fabrication of tissue-engineered rostral migratory streams and planar astrocyte cultures) was placed on the coverslip, and a TE-RMS was carefully extracted from its channel and laid onto the collagen-coated coverslip. Collagen was allowed to dry for 2–3 min prior to fixation. Following rinsing with PBS, cultures were permeabilized with 0.3% Triton X-100 and blocked with 4% normal horse serum for one hour at room temperature. Following rinsing with PBS, TE-RMS and planar astrocyte cultures were incubated in goat anti-glial fibrillary acidic protein (GFAP) antibody (1:1000) (Abcam, Cambridge, MA, USA; #ab53554, RRID: AB_880202) overnight at 4 °C. Cultures were then rinsed and incubated in donkey anti-goat 568 (1:500) (Thermo Fisher Scientific, Waltham, MA, USA; #A-11057, RRID: AB_2534104) and Hoechst solution (1:1000) (Thermo Fisher Scientific #H3570) in the dark for two hours at room temperature. Following secondary staining, cultures were rinsed with PBS, rinsed once with deionized water, then mounted onto glass slides with fluoromount G. The edges of the glass slides and coverslips were sealed with nail polish and allowed to dry in the dark prior to being stored at 4 °C.

### 2.5. Imaging

Phase-contrast images were obtained using a Nikon Inverted Eclipse Ti-S microscope, with digital image acquisition using a QiClick camera interfaced with Nikon Elements Basic Research software (4.10.01). Fluorescent images were obtained using a Nikon A1Rsi Laser Scanning Confocal microscope with either a ×60 or ×100 objective (CFI Plan Apo Lambda ×60 Oil, n.a. 1.40; ×100 Oil, n.a. 1.45).

### 2.6. RNA Extraction and RNA Sequencing

At DIV4 after fabrication, TE-RMSs were visualized with a phase-contrast microscope, and only TE-RMSs that were perfectly bundled—displaying tight bundles of longitudinally aligned astrocytes throughout the channel—were selected for RNA extraction (for additional TE-RMS selection details, see [38]). Bundled TE-RMSs were carefully extracted from their microchannels, immediately placed into RNAlater Solution (Invitrogen #AM7020), and stored at −20 °C (Figure 2F). Planar astrocyte cultures were visualized with a phase microscope to ensure culture integrity prior to RNA extraction.

Preserved TE-RMSs were pooled into one single sample (Figure 2G). Total RNA was extracted from TE-RMS samples and planar astrocyte samples simultaneously using the RNeasy Plus Universal Kit (73404, Qiagen, Hilden, Germany) (Figure 2H). The entire process was repeated 3 times (defined as Run 1, Run 2, and Run 3), facilitating RNA sequencing on 3 distinct TE-RMS and 3 distinct planar astrocyte samples. Quality of RNA was assessed via Bioanalyzer (Agilent, Santa Clara, CA, USA) automated electrophoresis, ensuring an RNA Integrity Number above 8.0 and the presence of 18S and 28S rRNA bands. Extracted RNA was reverse transcribed to complementary DNA, and the sequencing library was prepared using the SMART-Seq mRNA LP (634768, Takara Bio Inc., Kusatsu, Shiga, Japan), a non-stranded preparation for low amounts of total RNA. Sequencing was performed on an Illumina NextSeq 2000 (San Diego, CA, USA) to generate 100-base pair single-end reads with a minimum of 50 M reads per sample, which is sufficient to detect low-expression genes (Figure 2H).

### 2.7. Data Analysis

Fastp was used to qc and trim the sequencing fastq files, and Salmon was used to count the trimmed data against the transcriptome defined in Ensembl v111, which was built on the genome mRatBN7.2 [42,43]. Several Bioconductor (v3.18) packages in R (v4.33) were used for annotation and analysis [44]. The transcriptome count data were annotated and summarized at the gene level using tximeta and biomaRt [45,46]. Count data were analyzed in a paired structure, so that TE-RMSs and planar astrocytes from the same run were considered paired. Paired count data were analyzed using Principal Component Analysis (PCA), and plots were generated with PCAtools [47]. Heatmaps were generated using ComplexHeatmap [48]. Normalizations and statistical analyses were performed with DESeq2 [49]. The false discovery rate was calculated using the Benjamini–Hochberg procedure. Differentially expressed genes were defined as those with an adjusted *p*-value < 0.05. GSEA pathway analysis was conducted using the Molecular Signatures Database (MSigDB) on gene sets related to cytoskeleton and nuclear structure [50,51]. Analysis was performed on annotated genes only. Significance threshold was set at *p* < 0.05.

## 3. Results

To fabricate TE-RMSs, grids of hydrogel rectangular microchannels (Figure 1A,B) were dried and coated with extracellular matrix. Following complete polymerization and drying of the extracellular matrix, the microchannels were seeded with a dense suspension of astrocytes that bundled to form longitudinally aligned TE-RMSs (Figure 1C). This fabrication process, during which astrocytes self-assemble into a three-dimensional TE-RMS, induced profound morphological changes in the astrocytes compared to their counterpart astrocytes in planar culture. Specifically, cytoskeletal rearrangement and nuclear elongation were clearly evident in TE-RMS astrocytes. Phase-contrast microscopy and high-magnification confocal imaging revealed elongated cell nuclei and bidirectional intermediate filament processes in TE-RMSs (Figure 1E), while planar astrocyte samples had round nuclei and intermediate filament processes spread in all directions (Figure 1F–H).

**Figure 3:**
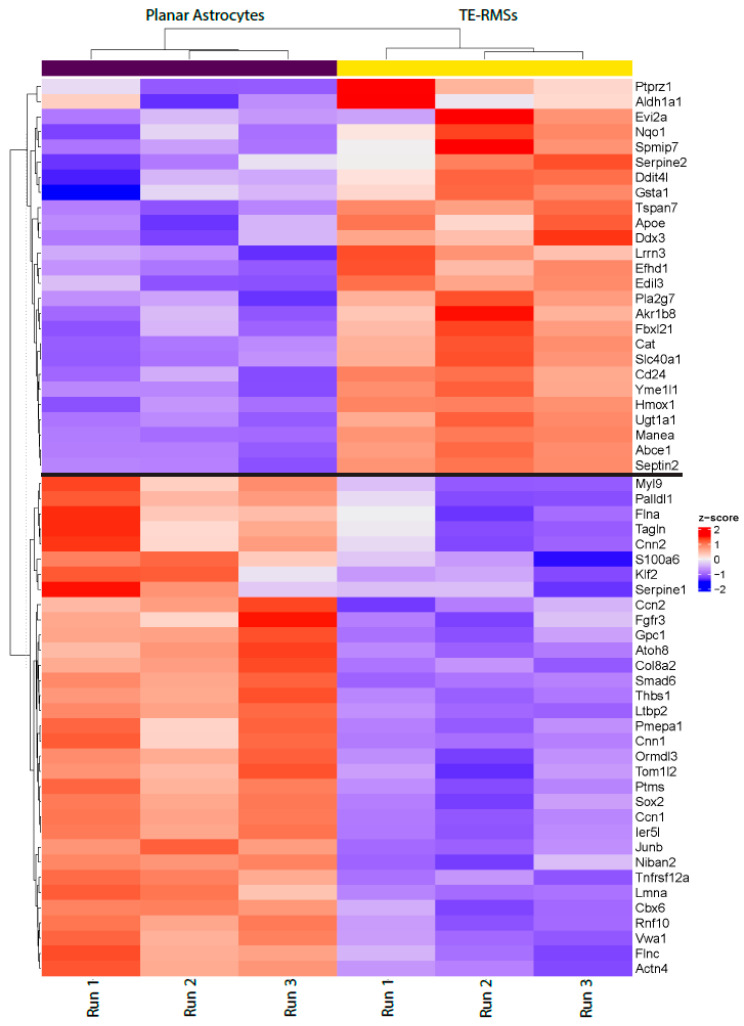
Heatmap with hierarchical clustering. Using normalized count data, we plotted a subset of genes in the upper portion of the graph that had the highest normalized counts for TE-RMSs, while a subset of genes in the lower portion of the graph had the highest normalized counts in planar astrocytes. Specimens are represented by columns, and each gene is represented by a row. Red indicates high expression, and blue indicates low expression. Through the tree diagram, hierarchical clustering demonstrated that TE-RMSs are closely associated with each other, and planar astrocytes are closely associated with each other. Additionally, there are many rows of highly expressed genes in the TE-RMS columns that may indicate gene candidates for future studies.

To begin evaluating transcriptomic changes in TE-RMS astrocytes compared to planar astrocytes, we pooled samples for each run, extracted total RNA, assessed RNA quality, and then conducted RNAseq. A total of 18,304 genes were detected with an approximately 60% alignment rate. This alignment rate, when paired with the >50 M reads per sample, is sufficient for gene-level differential expression, and the 100 bp read length provides sufficient detail for mapping and quantification. From the RNAseq output, we first generated a heatmap with hierarchical clustering using normalized count data of a subset of genes (Figure 3). This heatmap served as a high-level overview of highly expressed genes, and the hierarchical clustering tree diagram helped visualize similarities between specimens. The rows of genes in the upper half of the heatmap had the highest normalized counts for TE-RMS astrocytes, while the genes in the lower half had the highest counts for planar astrocytes. Additionally, the TE-RMS sample columns had many rows of highly expressed genes for all three runs, potentially indicating genes for future analysis. However, gene expression also varies between sample runs within an experimental group (for example, Aldh1ah, Evi2a, and Serpine1), indicating biological variability between sample runs. Through hierarchical clustering, we noted the clustering of all three TE-RMS runs with each other and the clustering of all 3 planar astrocyte runs with each other. This suggested that, even with sample run biological variability, the gene expression patterns of TE-RMSs were more similar to each other compared to planar astrocytes.

**Figure 4:**
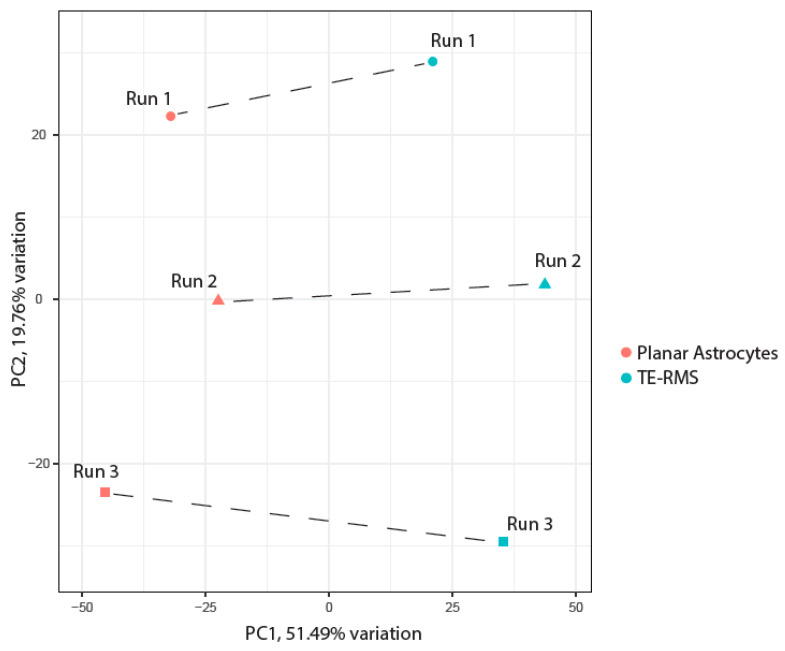
Principal component analysis. The first 2 principal components (PC) are labeled, followed by the percentage of variance explained by each PC. We visualized the close clustering of TE-RMS astrocytes (blue-green colored shapes) versus planar astrocytes (red colored shapes) along PC1 (51.49% variation), and the clustering of experimental runs along PC2 (19.76% variation). These PC analysis results suggested that PCs can differentiate TE-RMS astrocytes from planar astrocytes, as well as experimental runs to a lesser extent.

We continued our exploratory data analysis through PCA, which processed our large transcriptomic data set and transformed it into smaller sets that still contain the majority of the information. The result of this reduced data set complexity was a simplistic overview that demonstrated clear separation between our experimental groups. The first two PCs of the gene expression data explained over 70% of the variance (Figure 4). PC1 explained 51.49% and PC2 explained 19.76%. Along PC1, we saw a clear separation in our experimental groups as TE-RMS samples clustered on the right of the axis, while planar astrocytes clustered on the left side of the axis. Interestingly, PC2 separated our samples according to experimental run, as TE-RMS and planar astrocyte specimens from Run 1 clustered with each other, as Run 2 specimens clustered with each other, and Run 3 specimens clustered with each other. This indicated some gene expression differences between sample runs; however, this PC2 percent variation was smaller and less influential than PC1. Overall, our PCA results suggested that the transcriptomic expression data consolidated into PCs can differentiate TE-RMS astrocytes from planar astrocytes.

**Figure 5:**
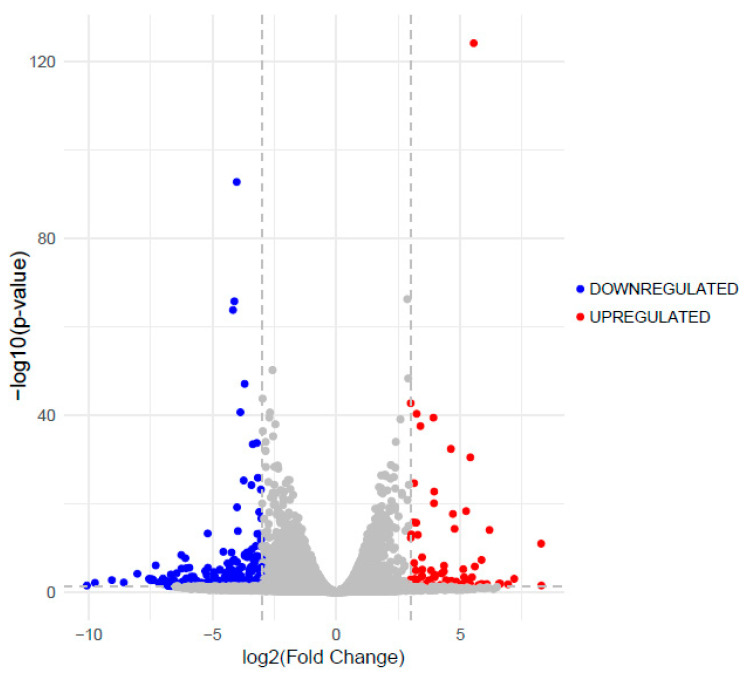
Differentially expressed genes in TE-RMS compared to planar astrocytes. The volcano plot depicts each gene’s −log_10_(*p*-value) and log_2_ fold change. To examine only significant and highly impactful genes, the horizontal dashed line indicates a false discovery rate adjusted *p*-value < 0.05, and the vertical dashed lines indicate a ±3 fold change. A total of 256 downregulated genes (blue dots) and 91 upregulated genes (red dots) met these criteria. All other genes are labeled with grey dots.

We performed differential expression (DE) analysis to determine which gene expression differences were statistically significant between TE-RMS astrocytes and planar astrocytes. Compared to planar astrocyte expression, 4008 genes were differentially expressed in TE-RMS astrocytes, with 2076 downregulated genes and 1932 upregulated genes (Figure 5). To examine only the most impactful genes, we applied a cutoff of ±3 fold change (log2), with 256 genes downregulated and 91 genes upregulated. Tables below present the top ten downregulated (Table 1) and upregulated (Table 2) genes (All DE results are available in Supplemental Table S1). We also conducted gene set analysis of Hallmark pathways to provide a broad functional overview of gene set changes between experimental groups (Supplemental Figure S1). Additionally, we examined our DE analysis results for *robo2* and *ezrin* expression, as we have previously reported that the endogenous RMS and TE-RMS astrocytes have enriched Robo2 and Ezrin proteins compared to non-RMS astrocytes in vivo or planar astrocyte cultures in vitro [12]. DE analysis revealed a non-significant increase (1.17 log2 fold) in Robo2 expression (*p* = 0.0791) and a significant increase (0.79 log2 fold) in Ezrin expression (*p* < 0.0001).

**Figure 6:**
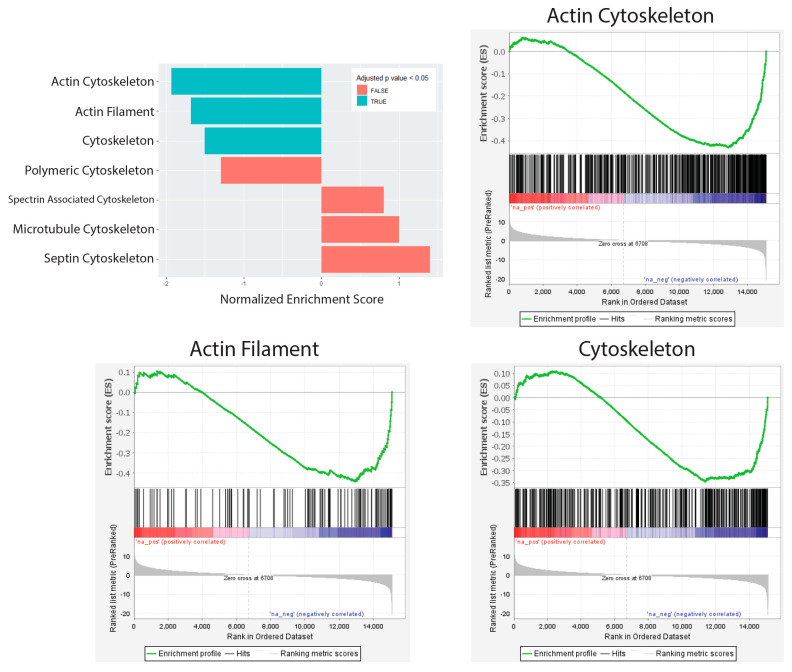
Cytoskeleton gene set analysis. Gene set analysis was conducted on cellular components related to cytoskeleton structure using the molecular signatures database annotated gene sets. Cellular components were significantly enriched for Actin Cytoskeleton (454 genes, NES = −1.930, adjusted *p*-value < 0.0001) (Supplemental Table S2), Actin Filament (106 genes, NES = −1.681, adjusted *p*-value = 0.0034) (Supplemental Table S3), and Cytoskeleton (332 genes, NES = −1.500, adjusted *p*-value = 0.0142) (Supplemental Table S4). Graphical representations of the rank metric score and the running enrichment score are presented for the Actin Cytoskeleton, Actin Filament, and Cytoskeleton gene sets. In the heat bar, red colors indicate upregulated genes and blue colors indicate downregulated genes.

**Figure 7:**
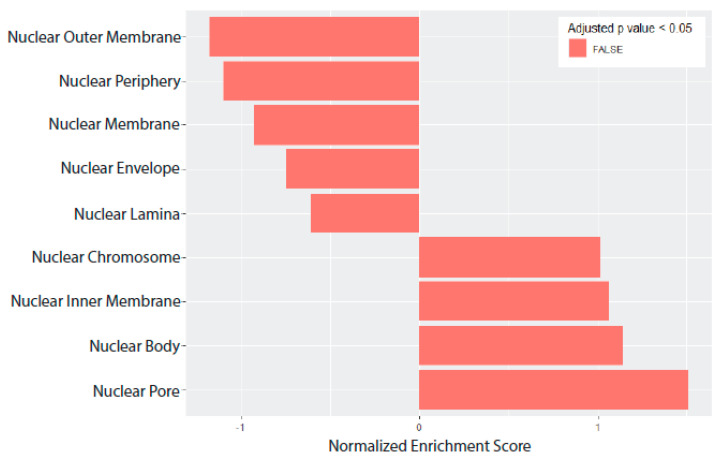
Nuclear gene set analysis. Gene set analysis was conducted on cellular components related to nuclear structure using the molecular signatures database annotated gene sets. No gene set was significantly enriched.

Our previous research has shown that the unique morphology of TE-RMS astrocytes mimicked the unique cytoskeletal and nuclear architecture of the endogenous rat RMS [37]. Therefore, we conducted gene set analysis on cellular component sets related to cytoskeleton (Figure 6) and nuclear (Figure 7) structure to identify gene expression changes that may underlie these morphological changes. Using the molecular signatures database, we assessed annotated gene sets for the following: Cytoskeleton, Actin Cytoskeleton, Actin Filament, Microtubule Cytoskeleton, Polymeric Cytoskeleton, Septin Cytoskeleton, Spectrin Associated Cytoskeleton, Nuclear Body, Nuclear Chromosome, Nuclear Envelope Lumen, Nuclear Inner Membrane, Nuclear Lamina, Nuclear Membrane, Nuclear Outer Membrane, Nuclear Periphery, and Nuclear Pore.

From the *p*-values of our DE analysis, we ranked all our genes and generated a local statistic called the rank metric score. This rank metric allowed us to sort our genes so that upregulated genes with small *p*-values were at the top of the list and downregulated genes with small *p*-values were at the bottom of the list. We also generated a running enrichment score for each gene by starting at the top of our ranked gene list and adding to the running sum if the gene is a member of the gene set of interest, or subtracting if the gene is not in that gene set. Finally, the enrichment score was the largest value (positive or negative) achieved during the running sum. This score is normalized depending on the gene set size and labeled as our normalized enrichment score (NES), which allowed direct comparison of all gene sets analyzed. We calculated cellular components that were significantly enriched for Cytoskeleton (332 genes, NES = −1.500, adjusted *p*-value = 0.0142) (Table 3), Actin Cytoskeleton (454 genes, NES = −1.930, adjusted *p*-value < 0.0001) (Table 4), and Actin Filament (106 genes, NES = −1.681, adjusted *p*-value = 0.0034) (Table 5) (Figure 6). No gene set was significantly enriched for nuclear structure (Figure 7).

## 4. Discussion

The TE-RMS is a tissue-engineered biological scaffold comprising aligned astrocytes, designed to facilitate NPC migration. Our previous work described morphological changes in astrocytes during their self-assembly into the TE-RMS, specifically cytoskeletal remodeling and nuclear elongation [37]. In the current study, we sought to examine the genetic underpinnings of these morphological changes through RNA sequencing. We found 256 downregulated genes and 91 upregulated genes with over a 3-fold change in TE-RMS astrocytes compared to planar astrocytes. Additionally, we hypothesized that TE-RMS astrocytes would have significant changes in cytoskeletal and nuclear gene sets compared to planar astrocytes, owing to their altered cytoarchitectural and nuclear morphology. Using gene set enrichment analysis, we found significant enrichment for cytoskeleton, actin cytoskeleton, and actin filament gene sets. However, we did not find significant enrichment for any nuclear structure-related gene sets, so we are compelled to partially reject our hypothesis.

RNA sequencing allowed us to map the complex cellular responses and characterize the molecular phenotype of the unique TE-RMS astrocytes. Our higher-level analyses—heatmaps with hierarchical clustering and PCA—demonstrated that TE-RMS astrocytes had observationally distinct expression profiles compared to planar astrocytes. PCA results appeared to partially differentiate between TE-RMS astrocytes and planar astrocytes and, to a lesser degree, the experimental run from which the astrocytes were isolated.

Yet it is through the differential expression analysis that we determined the individual genes that were significantly different between our experimental groups. One of our significantly downregulated genes was LOC690251 (−10.06 fold). LOC690251 is similar to a SUMO/sentrin-specific protease 5, which breaks down SUMO proteins involved in many post-translational modifications and may be required for cell division [52,53]. Downregulation of LOC690251 suggests that astrocyte cell division within the TE-RMS may be minimized, possibly due to the astrocytes prioritizing alignment, cell-cell adhesions, and expression of functional proteins. *ccn5* (−9.05 fold) is a cellular communication network factor relevant to the connective tissue growth factor family and involved in the regulation of cancer progression [54]. *lrfn1* (−8.02 fold), a family of adhesion molecules also known as synaptic adhesion-like molecules (SALMs), is involved in neurite outgrowth and synapse formation in neurons; however, its role in astrocytes has not been established [55]. Downregulation of *ccn5* and *lrfn1* may alter astrocyte adhesion to each other and the ECM, which may benefit the TE-RMS as astrocytes need to align in a bidirectional, tightly coupled pathway with specific adhesion patterns as opposed to the omnidirectional adhesion and communication typical of stellate astrocytes in the brain parenchyma.

One of the significantly upregulated genes included *lrrc45* (8.27 fold), which connects centrosomes during interphase of the cell cycle and organizes microtubules [56]. Centrosome cohesion may affect numerous cellular processes, including cell polarity, motility, and transport—suggesting an important influence on TE-RMS astrocyte motility, bipolar shape, and ability to divide if needed [57]. Another significantly upregulated gene is *cntn1* (7.18 fold), a cell surface adhesion molecule gene. When overexpressed in GFAP-expressing glioblastomas, the molecule creates a repellent effect on glioma cells, which may also play a role in TE-RMS cell organization and alignment [58]. Also, *cntn1* is implicated in inflammatory disorders and may facilitate crosstalk between astrocytes and microglia [59]. Notably, *pah* (6.62 fold) encodes for an enzyme that processes phenylalanine and alters astrocyte and microglia morphology in a *pah* variant-spliced mouse model [60]. Future experiments should assess if *pah*, along with *lrrc45* and *cntn1*, contribute to the bidirectional process filaments characteristic of TE-RMS astrocytes.

We also examined our DE analysis results for *robo2* and *ezrin* expression. Astrocytic Robo2 receptors ensure proper neuroblast migration in the RMS through chemorepulsion, while ezrin is a membrane-cytoskeletal protein expressed at high levels in RMS astrocytes [23,61,62]. We also previously reported that the astrocytes in the RMS in vivo and TE-RMS astrocytes in vitro both have more Robo2 and Ezrin than non-RMS astrocytes in vivo or planar astrocyte cultures in vitro [12]. Interfering with the function of these proteins impedes neuroblast migration through the RMS. In our current analyses, *ezrin* expression was significantly upregulated (*p* < 0.0001), while *robo2* expression was upregulated but narrowly missed the threshold for statistical significance (*p* = 0.0791). *ezrin* upregulation further validates our TE-RMS model with key characteristics of the endogenous rat RMS. While *robo2* expression was not statistically significant, other transcripts related to cell migration, such as *lrrc45* and *cntn1*, are greatly upregulated, suggesting that TE-RMS astrocytes express factors believed to facilitate neuroblast migration. Additionally, Robo2 receptors may undergo post-translational modification to function optimally in the RMS, which would not be reflected in the current study’s approach. Future studies should examine Ezrin and Robo2 gene and protein-level function over time in TE-RMS astrocytes.

Unlike our DE analysis, our gene set enrichment analysis allowed us to examine expression changes in functionally related gene sets. Using the molecular signatures database, we looked at all cellular component gene sets related to the cytoskeleton and nuclear structure and found significantly enriched gene sets for cytoskeleton, actin cytoskeleton, and actin filament gene sets, while no nuclear structure gene sets were significantly enriched. This may suggest that, while the angle of curvature in the agarose channels provided mechanical cues that signal gene expression changes related to cytoskeletal rearrangement, the nuclear elongation observed in RMS and TE-RMS astrocytes may result from cytoskeletal forces and not expression changes with nuclear structure genes. However, subtle nuclear transcriptomic changes could be missed due to statistical thresholds. Additional studies will be needed to differentiate between expression changes contributing to cytoskeletal rearrangement and those that resulted from cytoskeletal elongation, generating forces that act on the nucleus and potentially rearranging chromatin. Additional analyses should further explore chromatin remodeling and individual nuclear envelope-related transcripts to provide insight. Finally, gene expression changes are the primary driver for cell morphology alterations, but other factors, such as post-translational modifications and cellular component distribution, can also independently alter cell morphology.

Following the enrichment analysis, which determines whether an *a priori* defined set of genes shows statistical significance between two phenotypes, we identified over-represented genes within each significant gene set (Table 6). These are genes that are present more than would be expected in our selected gene set data. The over-represented genes in the cytoskeletal gene set were downregulated and include *flna*, which encodes for the actin-binding protein filamin A and serves as a versatile molecular scaffold [63]. Notably, *myl9* regulates myosin light chain, is highly expressed in astrocytes, and dramatically decreases after small molecule reprogramming of astrocytes into neurons [64]. Additionally, *palld* is involved in actin cytoskeleton organization, and its expression levels vary in astrocytes depending on their morphology [65]. Also, *myh9* is involved in actin-binding and crucial for adhesion and cell migration [66]. Finally, *synpo* encodes synaptopodin, an actin-binding protein that is typically found in postsynaptic densities and dendritic spines in neurons and may protect F-actin from disruption; however, its role in astrocytes is unknown [67].

Within the actin cytoskeleton and actin filament gene sets, we also see *flna*, *myl9*, and *palld* as over-represented genes that are downregulated. Additionally, *cnn1* encodes calponin 1, an actin filament-associated regulatory protein. Calponin isoforms *cnn2* and *cnn3* are expressed in neuronal tissue, while *cnn1* is typically found in smooth muscle cells, so its role in the current study is unclear [68,69]. Notably, *actn1* and *actn4* are alpha-actinin isoforms and belong to the spectrin gene superfamily, which is a group of cytoskeletal proteins [70]. Furthermore, *lmod1* is an actin-nucleating protein that has recently been discovered in astrocytes and neurons [71]. Finally, *tpm2* encodes beta-tropomyosin and is activated by alcohol and heat stress in astrocyte cultures [72].

Overall, these gene set analyses show the high degree of influence of structural protein-related genes that are downregulated in TE-RMS astrocytes. This is unsurprising as we have witnessed stark structural changes in TE-RMS astrocytes; planar astrocytes extend in all directions, while the aligned astrocytes of the TE-RMS take on a bidirectional morphology. Other literature has noted astrocyte cytoskeleton changes in aging astrocytes and during distress, conditions which produce atrophied or hypertrophied astrocytes [73]. These previously described morphologies are distinct from—and unlike—the bidirectional astrocytes of our TE-RMSs, which suggests adaptive remodeling of the cytoskeleton. Furthermore, astrocytic studies specifically focused on impaired actin dynamics note reduced morphological complexity as well as decreased astrocyte infiltration, which results in diminished axonal pruning [74,75].

While the current study was primarily descriptive, the data generated are useful to generate testable hypotheses in future studies. For instance, future studies could genetically alter these actin-related genes to study the effects on cell morphology and function—particularly nucleo–cytoskeletal interactions. Future studies could also test the efficacy of manipulating these genes to enhance the fabrication or function of the TE-RMS. Cell migration assays with and without genetically altered TE-RMSs would inform us of the optimal TE-RMS cytoarchitecture to improve its ability to facilitate neuroblast migration. Integrating proteomic analyses would allow us to verify the protein abundance of our DE genes and examine post-translational modifications. Furthermore, astrocytes exhibit regional and environment-dependent heterogeneity. Future studies should compare TE-RMS astrocytes to in vivo RMS astrocyte transcriptomes to validate the physiological relevance of our model. Finally, our TE-RMS samples were pooled to ensure sufficient RNA, yet this masks biological variability or outliers of individual TE-RMSs. The field is continuously advancing RNA isolation techniques, which we will employ in future studies when available.

## 5. Conclusions

Overall, the current study provides a detailed transcriptomic evaluation of a unique astrocyte-based engineered microtissue compared to planar counterparts. TE-RMS astrocytes demonstrated extensive actin-related gene expression changes, paired with histologically observed cytoskeletal rearrangement and significantly altered nuclear shape. This study highlights the TE-RMS as a novel platform to investigate factors underlying cytoskeletal arrangement, nucleo–cytoskeletal interactions, astrocyte function, and cell migration. Our findings offer an array of genetic targets that can be manipulated to systematically test the underlying mechanisms and/or augment these functions to enhance the efficacy of these living regenerative scaffolds.

## Figures and Tables

**Table 1:** Top ten downregulated genes with the greatest fold change (log2).

Gene Name	Description	Fold Change (log2)	Adjusted *p*-Value
*LOC690251*	similar to SUMO/sentrin specific protease 5 [Accession Number:1582860]	−10.06	0.0358
*wbp4-ps1*	WW domain binding protein 4, pseudogene 1 [Accession Number:2320129]	−9.73	0.0088
*ENSRNOG00000067245*	Unknown	−9.17	1.135 × 10^−7^
*ccn5*	cellular communication network factor 5 [Accession Number 621867]	−9.05	0.0021
*LOC685989*	hypothetical protein LOC685989 [Accession Number:1587786]	−8.57	0.0074
*lrfn1*	leucine rich repeat and fibronectin type III domain containing 1 [Accession Number:1304707]	−8.02	7.813 × 10^−5^
*hs3st3b1*	heparan sulfate-glucosamine 3-sulfotransferase 3B1 [Accession Number:1307326]	−7.55	0.0011
*xkr4*	XK related 4 [Accession Number:1549780]	−7.49	0.0022
*lypd8*	Ly6/Plaur domain containing 8 [Accession Number:1586251]	−7.46	0.0011
*LOC102549173*	histone H3.2-like [Accession Number:7709329]	−7.40	0.0016

**Table 2:** Top ten upregulated genes with the greatest fold change (log2).

Gene Name	Description	Fold Change (log2)	Adjusted *p*-Value
*lrrc45*	leucine rich repeat containing 45 [Accession Number:1590053]	8.27	0.0343
*cym*	chymosin [Accession Number:708486]	8.26	1.1964 × 10^−11^
*cntn1*	contactin 1 [Accession Number:621300]	7.18	0.0010
*abca17*	ATP-binding cassette, subfamily A (ABC1), member 17 [Accession Number:1560494]	6.93	0.0189
*pah*	phenylalanine hydroxylase [Accession Number:3248]	6.62	0.0137
*arhgef37*	Rho guanine nucleotide exchange factor 37 [Accession Number:1560471]	6.56	0.0137
*lrrc25*	leucine rich repeat containing 25 [Accession Number:1564818]	6.18	9.820 × 10^−15^
*itga2*	integrin subunit alpha 2 [Accession Number:621632]	6.07	0.0164
*ppfia2*	PTPRF interacting protein alpha 2 [Accession Number:1305021]	5.99	0.0251
*col10a1*	collagen type X alpha 1 chain [Accession Number:2371]	5.86	0.0154

**Table 3:** Top 5 over-represented genes in the Cytoskeletal gene set that are downregulated.

Gene Name	Description	Rank in Gene List	Rank Metric Score	Running Enrichment Score
*flna*	Filamin A [Accession Number: HGNC:3754]	15,098	−13.501	0.0006
*myl9*	Myosin light chain 9 [Accession Number: HGNC:15754]	15,097	−13.221	−0.0183
*palld*	Paladin, cytoskeletal associated protein [Accession Number: HGNC:3754]	15,084	−10.851	−0.0360
*myh9*	Myosin heavy chain 9 [Accession Number: HGNC:7579]	15,033	−8.190	−0.0479
*synpo*	Synaptopodin [Accession Number: HGNC:30672]	15,023	−8.037	−0.0587

**Table 4:** Top 5 over-represented genes in the Actin Cytoskeletal gene set that are downregulated.

Gene Name	Description	Rank in Gene List	Rank Metric Score	Running Enrichment Score
*cnn1*	Calponin 1 [Accession Number: HGNC:2155]	15,101	−13.986	0.0004
*flna*	Filamin A [Accession Number: HGNC:3754]	15,098	−13.501	−0.0132
*myl9*	Myosin light chain 9 [Accession Number: HGNC:15754]	15,097	−13.221	−0.0265
*actn4*	Actinin Alpha 4 [Accession Number: HGNC:166]	15,085	−10.932	−0.0388
*palld*	Paladin, cytoskeletal associated protein [Accession Number: HGNC:3754]	15,084	−10.851	−0.0495

**Table 5:** Top 5 over-represented genes in the Actin Filament gene set that are downregulated.

Gene Name	Description	Rank in Gene List	Rank Metric Score	Running Enrichment Score
*flna*	Filamin A [Accession Number: HGNC:3754]	15,098	−13.501	0.0006
*palld*	Paladin, cytoskeletal associated protein [Accession Number: HGNC:3754]	15,084	−10.851	−0.0480
*actn1*	Actinin Alpha 1 [Accession Number: HGNC:163]	15,042	−8.538	−0.0851
*lmod1*	Leiomodin 1 [Accession Number: HGNC:6647]	15,019	−7.929	−0.1149
*tpm2*	Tropomyosin 2 [Accession Number: HGNC:12011]	15,000	−7.515	−0.1428

**Table 6:** Function and role of over-represented genes in cytoskeletal and actin filament sets.

Gene Name	Biological Function and Role	References
*flna*	Molecular scaffold	[63] Feng & Walsh, 2004
*myl9*	Highly expressed in astrocytes Decreases after small molecule reprogramming	[64] Ma et al., 2019
*palld*	Actin cytoskeleton organization Expression in astrocytes vary depending on morphology	[65] Boukhelifa et al., 2003
*myh9*	Actin-binding Crucial for adhesion and cell migration	[66] Asensio-Juárez et al., 2020
*synpo*	Found in postsynaptic densities and dendritic spines May protect F-actin from disruption	[67] Okubo-Suzuki et al., 2008
*cnn1*	Actin filament-associated regulatory protein Typically found in smooth muscle, while other isoforms are found in neuronal tissue	[68] Liu & Jin., 2016 [69] Sankar et al., 2024
*actn1*	Alpha-actin isoform in the spectrin cytoskeleton family	[70] Murphy & Young, 2015
*actn4*	Alpha-actin isoform in the spectrin cytoskeleton family	[70] Murphy & Young, 2015
*imod1*	Actin nucleating protein in astrocytes and neurons May play a role in pathogenesis	[71] Nauen et al., 2020
*tpm2*	Beta-tropomyosin Activated by alcohol and heat-stress	[72] Pignataro et al., 2013

## Data Availability

The data that support the findings of this study are openly available in the Gene Expression Omnibus at https://www.ncbi.nlm.nih.gov/geo/query/acc.cgi?acc=GSE309109 (accessed on 22 September 2025), reference number [GSE309109]. Differential expression analysis and gene set enrichment analysis results are uploaded as supplemental material.

## Supplementary Materials

The following supporting information can be downloaded at [https://www.mdpi.com/article/10.3390/cells14211646/s1]. Supplemental Figure S1. Hallmark pathway analysis. Gene set analysis was conducted on Hallmark pathways using the molecular signatures database annotated gene set. Supplemental Table S1. Differential expression analysis. Differential expression analysis results comparing TE-RMSs to planar astrocytes. Supplemental Table S2. Actin cytoskeleton. Gene set enrichment analysis results for the actin cytoskeleton. Supplemental Table S3. Actin filament. Gene set enrichment analysis results for actin filament. Supplemental Table S4. Cytoskeleton. Gene set enrichment analysis results for the cytoskeleton.

## Abbreviations

The following abbreviations are used in this manuscript:

SVZ	Subventricular Zone
NPC	Neural Precursor Cell
RMS	Rostral Migratory Stream
TE-RMS	Tissue Engineered-Rostral Migratory Stream
RNA	Ribonucleic Acid
DIV	Days In Vitro
GFAP	Glial Fibrillary Acidic Protein
PCA	Principle Component Analysis
DE	Differential Expression
NES	Normalized Enrichment Score
