# Supplementary figures and images for: Astrocyte Transcriptomics in a Three-Dimensional Tissue-Engineered Rostral Migratory Stream
Source: Cells. 2025 Oct 22;14(21):1646. doi: 10.3390/cells14211646 (PMC12607521; doi:10.3390/cells14211646)

Hallmark Pathways NES from GSEA

Pathway

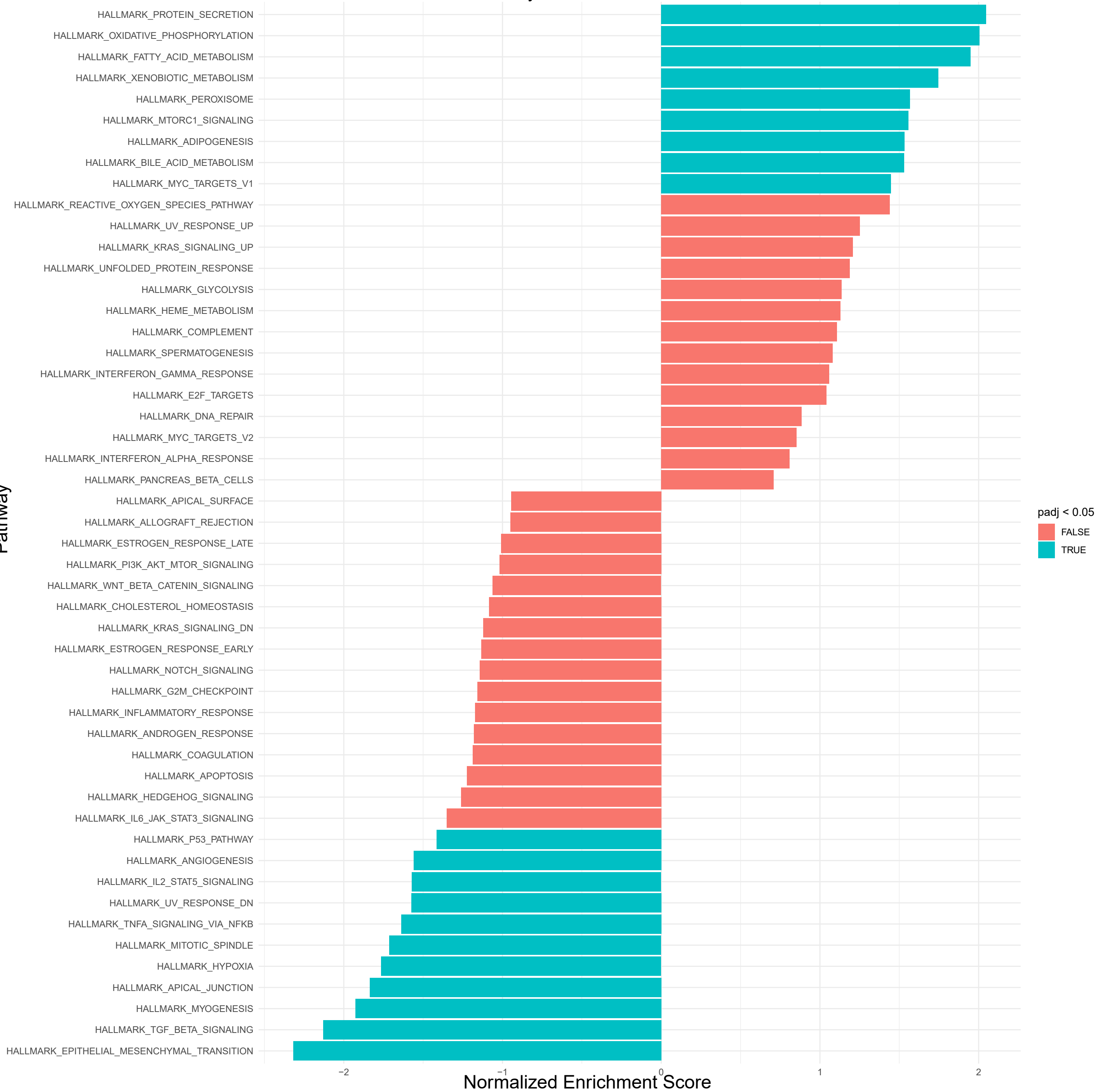

Supplement: Supplementary file 1 [file cells-14-01646-s001.zip › Supplemental Figure 1.pdf]
